# Idiopathic atrophic glossitis as the only clinical sign for celiac disease diagnosis: a case report

**DOI:** 10.1186/1752-1947-6-185

**Published:** 2012-07-04

**Authors:** Matteo Erriu, Fernando Canargiu, Germano Orrù, Valentino Garau, Caterina Montaldo

**Affiliations:** 1 Dipartimento di Chirurgia e Scienze Odontostomatologiche, Università degli studi di Cagliari, Via Binaghi 4, Cagliari, CA, 09121, Italy

**Keywords:** Atrophic glossitis, Celiac disease, Early diagnosis

## Abstract

**Introduction:**

The aim of this case report is to show how an oral condition, such as atrophic glossitis, can be the only clinical sign that allows an early diagnosis of celiac disease.

**Case presentation:**

Atrophic glossitis was detected by a dentist during a first routine examination of the oral cavity of a 17-year-old Sardinian young woman and then differential diagnosis was carried out to identify the etiology of her tongue condition. Considering the high prevalence of celiac disease in the patient’s birth area, the clinician took a blood sample to search for vitamin deficiency and immunological anomalies typically linked to celiac disease. Positive blood sample results allowed the patient to be referred to a gastroenterologist in order to perform a small intestine biopsy. The biopsy showed a strong atrophy of the intestinal villus so that it was possible to make a sure diagnosis of celiac disease. After five months on a gluten-free diet, the oral clinician was not able to find any signs of atrophic glossitis.

**Conclusions:**

Two important conclusions can be reached from this case report; first, the fundamental role played by the oral condition alone in finding and highlighting atypical forms of celiac disease and second, the importance of investigating systemic anomalies, in cases where there is a tongue condition such as atrophic glossitis and when it is impossible to identify local causes.

## Introduction

Tongue diseases could be reflections of altered systemic conditions or, also, initial forms of local and often severe pathologies (for example, carcinomas) [1,2]. The most commonly encountered tongue diseases resulting from systemic conditions are median rhomboid glossitis, atrophic glossitis, fissured tongue, and geographic tongue, while among local conditions, there are papilloma, hairy tongue and leukoplakia with their possible malignant evolution [2,3].

Atrophic glossitis (AG) is an inflammatory disorder of the tongue mucosa that shows a smooth, glossy appearance with a red or pink background [2]. The smooth appearance is linked to the atrophy of filiform papillae that causes the development of circinate erythematous ulcer-like lesions of the dorsum and the lateral border of the tongue [2,4]. This disease is primarily related to various conditions such as amyloidosis, chemical irritations, drug reactions, local infections such as candidiasis, nutritional deficiencies, pernicious anemia, malnutrition, sarcoidosis, Sjögren’s syndrome, systemic infections, psoriasis, vasculoerosive diseases or celiac disease [1,2,4,5]. Since there are so many different possible causes, etiological identification of AG can be extremely difficult, so that various analyses and investigations are needed before diagnosis [1]. For the same reason, the etiology sometimes remains unknown until symptoms other than tongue inflammation are identified [4].

Celiac disease (CD) is a disorder linked to an autoimmune intolerance to gliadin, a protein contained in gluten. The main target of this intolerance is the mucosa of the small intestine with development of histological lesions characterized by various degrees of villous atrophy, crypt hyperplasia, damage to the surface epithelium, and an increased number of activated lymphocytes and other inflammatory cells infiltrating the lamina propria.

CD diagnosis is based on observation of systemic clinical signs followed by blood analysis and is finally determined by a histopathological examination of the small intestine. Today, the greatest difficulty in obtaining this diagnosis is the identification of clinical signs that are not ‘typical’ for CD so that many patients never arrive at the gastroenterological examination stage. CD can be divided into different clinical forms known as classical, atypical, subclinical and latent. The classical form is characterized by intestinal symptoms such as chronic diarrhea, weight loss, growth deficit and vomiting; all other symptoms from other sites determine the atypical, subclinical and latent forms [6]. Clinical signs could also be divided into two groups of direct or indirect symptoms [7]. Direct symptoms are directly linked to the immune disorder, while indirect symptoms are the consequence of the intestinal damage that causes poor absorption of nutrients in the cells of the basal layer.

Once the disease is suspected through the identification of these clinical signs, serological analysis is necessary to confirm this diagnosis. Principal serological markers used for CD diagnosis are the tissue transglutaminase (tTG) antibody test and the IgA-endomysial antibody (EmA) test, while other tests such as antigliadin (AGA) or antireticulum (ARA) are no longer routinely performed [8]. Another serological test often performed when CD is suspected is the HLA-DQB1 haplotype. HLA typing is an important marker for demonstrating the possibility that a patient is affected by CD. Interest is increasing in the link between the development of the different clinical forms of CD and the different HLA-DQB1 genotypes [9,10]. Recently more and more importance has been given to the link between this gene expression and the development of atypical and latent forms [10].

Early CD diagnosis is indispensable for avoiding long term effects linked to untreated pathology. Various reports in the literature describe how patients with a never-identified CD showed an increasing incidence of small bowel malignancies, adenocarcinoma and enteropathy-associated T-cell lymphoma [7].

The aim of this case presentation is to highlight how AG could be the only previous sign that can be used to suspect a gluten intolerance and how it is always necessary to identify tongue inflammation etiology.

## Case presentation

A 17-year-old Sardinian young woman underwent an initial examination at the dentist’s office during a routine checkup in 2010. After obtaining informed consent from her parents, clinical evaluation was performed, during which some caries were detected and also the need for an orthodontic consultation. The presence of an atrophy of filiform papillae with circinate erythematous ulcer-like lesions of the dorsum and the lateral border of the tongue was observed.

During the anamnesis, neither the patient nor her parents reported any history of systemic disease or, in particular, any gastrointestinal symptoms. Talking with the patient and her parents, it was possible to verify that the tongue affection had started when she was five years old with no other symptoms and with intermittence. During her past medical history, no one had ever investigated these tongue lesions.

With these elements, the clinician identified the tongue condition as an AG and started with a more detailed analysis of systemic and local conditions related to this pathology. The patient, as highlighted by the anamnesis, did not show any other symptoms or signs related to pathologies typically linked to an AG, apart from her short stature [8]. She was only 145 cm tall but this condition had previously gone unnoticed because her parents were also short. Considering the presence of short stature with an AG, the problems could be related to a nutritional dysfunction such as vitamin B12 deficiency. Given that Sardinia is an area with a high frequency of CD, gluten enteropathy became the first suspect as a possible cause for the eventual vitamin B12 deficiency.

As a first step, a blood sample was requested to determine the vitamin deficiency and to perform AGA and tTG antibody tests and the EmA test. Hematologic tests showed a vitamin deficiency with positive results for antibody tests (Table 1) so a small intestine biopsy was performed during a gastroenterological consultation [7].

**Table 1:** Blood sample test analysis show as suspected a B12 vitamin deficiency, immunological analysis showed a positive response for tissue transglutaminase and IgA-endomysial antibody

**Test**	**Normal range**	**Patient’s value**
Folic Acid	3 to 17 ng/ml	1.65
Vitamin B12	193 to 982 pg/ml	<150
Antigliadin (IgG)	---	Positive
Antigliadin (IgA)	---	Negative
transglutaminase (IgA)	---	Positive
IgA-endomysial antibody (EmA)	---	Positive

A diagnosis of CD was made based on the findings from the biopsy sample; such as: the characteristic changes in intraepithelial lymphocytosis, crypt hyperplasia and a Marsh type IIIc villous atrophy. She was treated by excluding gluten-based-food from her diet (gluten-free diet). After five months she repeated the intraoral examination where it was possible to verify remission of the AG.

## Discussion

More than 100 years after its first description in 1887, CD is still an orphan pathology, since any specialist can be the sole person responsible for its diagnosis while, at the same time, collaboration among more specialists is indispensable for the recognition of early symptoms of CD, especially for atypical forms.

Today, this is a common pathology with an incidence of around one in 300 people worldwide and a higher prevalence in some areas, such as Sardinia in Italy where the incidence is one in 100 people. The greatest diffusion is reported among patients with other autoimmune diseases such as type 1 diabetes and Hashimoto’s thyroiditis with a frequency three to 10 times higher than in the general population [7].

Year after year, the discovery of this celiac iceberg has assumed greater importance with regard to obtaining adequate prevention of CD complications, so that it has become clear how clinical manifestations of this pathology are sometimes insidious and hard to discover or recognize. For this reason many medical specialists other than gastroenterologists, such as neurologists, dermatologists, gynecologists and dentists have also started to analyze their role in providing a suspected diagnosis of this pathology [6,7,10]. This attention to CD aims at allowing the recognition of its symptoms without any delay between early sign detection and CD diagnosis, a time during which the patient often receives many different wrong diagnoses before that of CD . For this reason, oral signs linked to CD, described by many scientific works over the past few years, have shown that oral anomalies, such as dental enamel defects and recurrent aphthous stomatitis, could have a prevalence of 60% in association with CD [10].

## Conclusions

Abnormalities of the tongue can represent an unclear pathology for the dentist, especially when they are linked to autoimmune diseases or a nutritional deficiency. This case study has tried to show as clearly as possible how tongue diseases could be mirrors for systemic conditions and not only for some local alterations. Different kinds of glossitis can be linked to systemic pathology usually not linked to common dental practice [2]. For this reason, the clinician, after excluding local etiology through a differential diagnosis, simply ignores the disease instead of performing a more accurate analysis. On the contrary, this paper has demonstrated that the dentist must always work to discover the cause of tongue affection and that this goal can be achieved by working together with other specialists [2]. In particular, it has been shown how starting from an AG, the dentist in collaboration with the gastroenterologist, can have a fundamental role in CD diagnosis. As previously recalled, when CD is not identified and the patient does not follow a gluten-free diet, he or she can be exposed to severe intestinal complications.

In conclusion, in the presence of a tongue lesion of uncommon etiology, patients must always be referred to a dentist, highly qualified in oral pathology, for a diagnosis to be obtained.

## Consent

Written informed consent was obtained from the patient’s legal guardian for publication of this case report and any accompanying images. A copy of the written consent is available for review by the Editor-in-Chief of this journal.

## Competing interests

The authors declare that they have no competing interests.

## Authors’ contributions

ME performed the first examination, the diagnosis and wrote the paper. FC and VG were major contributors for differential diagnosis, GO and CM were major contributors in blood sample interpretation and in writing the manuscript. All authors read and approved the final manuscript.
